# Streptococcus gordonii Empyema: A Rare Presentation of Streptococcus gordonii Infection

**DOI:** 10.7759/cureus.4611

**Published:** 2019-05-07

**Authors:** Hafsa Farooq, Tayyaba Mohammad, Amna Farooq, Qasim Mohammad

**Affiliations:** 1 Internal Medicine, Waterbury Hospital, Waterbury, USA; 2 Internal Medicine, Rutgers New Jersey Medical School, Jersey City, USA; 3 Miscellaneous, Shaikh Khalifa Bin Zayed Al-Nahyan Medical and Dental College, Lahore, PAK; 4 Internal Medicine, Windsor University School of Medicine, Boonton, USA

**Keywords:** strep gordonii, empyema, streptococcus gordonii

## Abstract

Empyema is often caused by Streptococcus pneumoniae, Staphylococcus aureus, and a variety of gram-negative organisms as well as anaerobes. Streptococcus gordonii (S. gordonii) is among some of the initial colonizers of the periodontal environment that is recognized to cause bacterial endocarditis. However, there are only a few case reports of S. gordonii causing empyema in the literature. We report the case of a 75-year-old male who presented with coughing up blood-tinged sputum. Physical examination revealed decreased breath sounds in the right lung base. Chest X-ray demonstrated a lower, right-sided, loculated pleural effusion. He underwent ultrasound-guided chest tube placement. The pleural fluid culture grew S. gordonii. He was started on ampicillin/ sulbactam. The follow-up computed tomography (CT) scan showed no significant improvement. Given his inability to improve with antibiotics and chest tube drainage, he was referred to an advanced care center for decortication of lung tissue.

## Introduction

For many years, Streptococcus (S.) pneumoniae, S. aureus, S. pyogenes, and some anaerobes have been studied to be the leading pathogens associated with empyema. Viridans streptococci, including Streptococcus gordonii, are anaerobic, gram-positive organisms that normally inhabit the oral tract and are often associated with dental plaque formation. This bacterium has been established as one of the main causative organisms in the development of subacute bacterial endocarditis (SBE) [1]. However, the literature on the implications of these organisms in other infections, such as empyema, is limited.

## Case presentation

**Figure 1:**
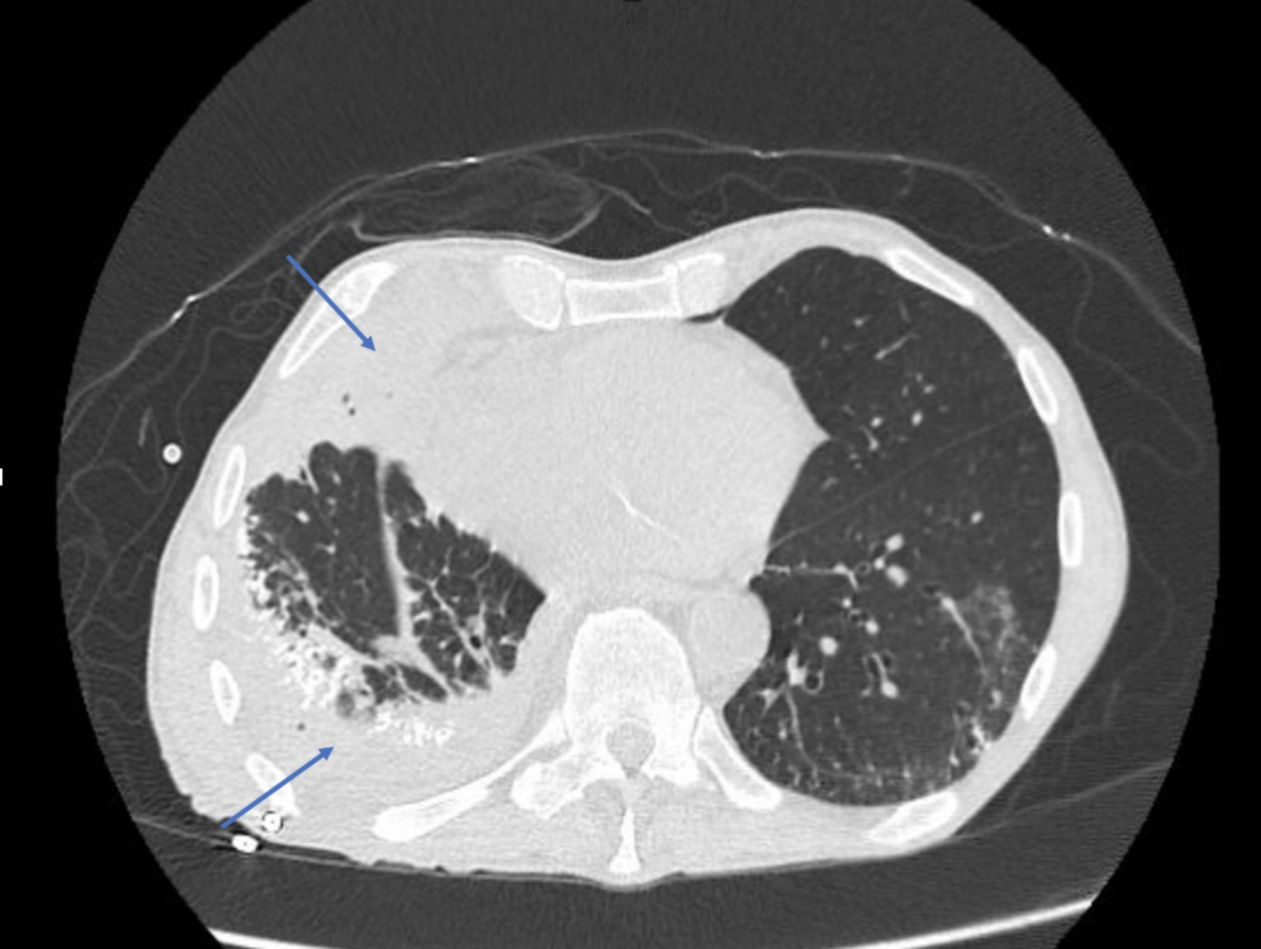
Loculated Pleural Effusion: Empyema

A 75-year-old male with a history of tobacco abuse and depression was admitted to a community hospital due to three weeks of fatigue and two days of coughing up blood-tinged sputum. The physical examination was significant for decreased breath sounds on the right lung base and normal oral cavity with no cervical lymphadenopathy. The initial laboratory evaluation was normal and did not reveal leukocytosis. Blood cultures were negative. Human immunodeficiency virus (HIV) test was non-reactive. Chest radiograph revealed a large, lower, right-sided loculated effusion (Figure 1). He underwent ultrasound (US)-guided chest tube placement, which yielded 200 ml of exudative fluid with 34,300/mm^3^ nucleated white blood cells (1372/mm^3^ granulocytes). Pleural fluid was sent for laboratory analysis. He was started on intravenous (IV) ampicillin/sulbactam. Pleural fluid culture yielded a rare species, S. gordonii. During his stay at the hospital, he was noticed to have mild dysphagia with regular diet. Aspiration was thought to be the reason for his infection. Repeat chest X-ray showed a worsening of empyema. tPA was administered in the chest tube, which yielded 550 cc of the exudative fluid. Follow-up CT chest revealed loculated effusions. Due to failed chest tube drainage with tPA and no improvement in the loculated effusions with antibiotics, he was transferred to a more advanced center for video-assisted thoracoscopic (VATS) decortication.

## Discussion

Thoracic empyema is an infection of the pleural space that commonly occurs as a complication of pneumonia, malignancy, tuberculosis, or thoracic trauma. A pleural infection comprises empyema when presenting with frank pus in the pleura and complicated parapneumonic effusions in the absence of pus but with other signs of infection [2]. Early recognition and drainage are critical to the patient’s survival and care. Many different types of bacteria may cause pneumonia leading to empyema, but the two most common are Streptococcus pneumoniae and Staphylococcus aureus. Our patient presented with empyema from a very unusual organism, S.gordonii.

S. gordonii is an anaerobic, gram-positive, non-motile coccus that is a member of the viridans group. S. gordonii is an inhabitant of the oral flora where it is the principal etiological agent of dental caries, causing the dissolution of tooth enamel by the acid end products resulting from carbohydrate metabolism [3]. This bacterium is capable of spreading to extraoral sites, causing systemic infection [1]. Viridans streptococci, such as S. gordonii, along with S. sanguinis and S. oralis, are commonly found in the blood cultures of patients with infective endocarditis [4]. S. gordonii is capable of colonizing platelet-fibrin thrombi formed on abnormal heart valves or the endocardium, thus it is frequently associated with causing subacute bacterial endocarditis [1]. However, there are only a few case reports of S. gordonii empyema reported in the literature.

The diagnosis of empyema is important for the rapid initiation of effective pleural drainage and appropriate antibiotic therapy. The optimal evaluation of empyema requires a contrast-enhanced chest CT. The criteria for thoracentesis is a thickened parietal pleura on a contrast-enhanced CT scan, a finding suggestive of empyema [5]. Ultrasonography of the pleural space allows for the characterization of pleural fluid collections with septations and loculations. The pleural fluid should be sent for a microbiologic analysis, cell count with differential, and chemistries.

Management and treatment options for thoracic empyema include systemic antibiotic therapy, adequate pleural fluid drainage, and obliteration of the empyema [6]. Tube thoracostomy, video-assisted thoracoscopic surgery (VATS), open decortication, and open thoracostomy are the drainage options for empyema. Adequate pleural fluid drainage is achieved when repeat imaging through a chest CT demonstrates the disappearance of any residual loculations [6].

## Conclusions

S. gordonii is an anaerobic, gram-positive member of the viridans group Streptococci and is a natural inhabitant of the oral cavity. This report illustrates a rare case of thoracic empyema caused by S. gordonii. Our patient presented with a loculated effusion with the characteristics of empyema, which failed to resolve after antibiotic treatment, multiple drainage, and tPA administration. The patient was transferred to a more advanced center for VATS decortication.
